# Organization of Cooperating Aluminum Pairs in Ferrierite Evidenced by Luminescence Quenching

**DOI:** 10.1021/acs.jpcc.3c00585

**Published:** 2023-04-07

**Authors:** Joanna E. Olszówka, Pavel Kubat, Jiri Dedecek, Edyta Tabor

**Affiliations:** J. Heyrovský Institute of Physical Chemistry of the Czech Academy of Sciences, Dolejškova 2155/3, 18200 Prague, Czech Republic

## Abstract

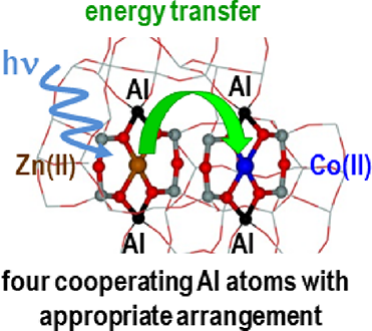

We show that four cooperating Al atoms located at the two neighboring six-membered (6-MR) rings in the ferrierite framework can be readily discerned by luminescence studies. Thus, luminescent Zn(II) cations accommodated by one aluminum pair of the 6-MR ring can be effectively quenched by neighboring Co(II) ions stabilized by the second ring. Quenching occurs via the energy transfer mechanism and allows estimation of the critical radius of Zn(II)–Co(II) interactions. This points to the appropriate geometry and distance of the transition metal ions accommodated within zeolite, providing direct evidence of the four-aluminum atom arrangement in the ferrierite framework.

## Introduction

Catalytic activation of molecular oxygen for hydrocarbons’ transformation reactions to value-added chemicals is desirable for future industrial processes. Such an approach can help significantly reduce the environmental burden caused by currently used oxidants, e.g., strong peroxy/acids, aiming at more atom-efficient and waste-free technologies.^1^ It was shown that the bare cations of Fe, Ni, Mn, and Co embedded in zeolitic matrices (CHA, ZSM-5, or FER) can stabilize active oxygen forms originating from the splitting of N_2_O or O_2_.^2−12^ This active oxygen form called α-oxygen ([M^2+^ = O^2–^]^2+^) exhibited high oxidation properties toward the transformation of methane to methanol or benzene to phenol.^2,5,6^ The employment of O_2_ as a cheap and eco-friendly oxidant of hydrocarbons attracts great attention from both scientific and industrial communities. Oxygen-containing products formed after the O_2_ oxidation of hydrocarbons can be used as platform chemicals to produce various other value-added products. Methanol obtained from methane is considered one of the most promising alternative energy vectors that can be employed to replace fossil fuels in the near future. However, methane, a stable molecule with low polarizability and four high-energy C–H bonds, is difficult to oxidize and requires reactive oxygen forms stabilized on heterogeneous catalysts.^2^

Recently discovered in crystalline aluminosilicate zeolitic matrix, distant binuclear transition metal ion (TMI) sites provide a promising solution as they are able to split molecular oxygen, forming the pair of two α-oxygen species ([M^2+^ = O^2–^]^2+^).^3−6^ Their activity was already reported in the oxidation of methane,^7,8^ revealing their great potential in oxidation chemistry.

Binuclear TMI sites are formed by two cooperating M(II) cations (M = Fe, Co, Mn, Ni) capable of the M(II) ↔ M(IV) redox cycle required for the activation of molecular oxygen (the four-electron process). For their formation, two requirements must be met simultaneously:

(i) Proper topology of the zeolite matrix resulting in the presence of two opposite zeolite rings (typically six-member rings) forming cationic sites in distances of ca. 6.5–8.0 Å. Only M(II) cations facing each other at the proper distance, given by the distance of zeolite rings, can split molecular oxygen.^4^

(ii) Cooperation of four Al atoms isomorphously substituted to the zeolite framework in a specific arrangement—two Al atoms in two opposite rings.

In zeolites, isomorphous substitution of an aluminum framework to a silicate framework results in a framework negative charge, which is compensated by extra-framework cationic species, e.g., in the form of divalent TMI.^9^ While appropriate topology for the formation of distant TMI sites can be assessed by the combination of structural information supported by DFT calculations,^3−5^ suitable Al organization in the zeolite framework cannot be predicted. The already developed methodology for the analysis of Al atoms distribution encompasses multi-spectroscopic studies and needs to be adjusted to the particular zeolitic structure. For Si-rich zeolites, isolated Al atoms (able to accommodate only monovalent cations in extra-framework sites, e.g., Cu(I)), and Al pairs (Al–(Si–O)_2_–Al sequences in one ring, responsible for the accommodation of M(II) in extra-framework sites) were reported.^9,10^ The formation of binuclear TMI sites requires not only the accommodation of divalent cations but also their location in two opposite rings. Two Al atoms (Al pairs) in the ferrierite matrix are located in three extra-framework cationic sites: α-, β-, and γ-sites. The α-site is situated in the main channel with each of the two Al atoms in one 6M ring, the β-site is located in a 6M ring in an 8M channel, and the γ-site is a complex boat-shaped structure.^9,11^ Due to such arrangements of the sites able to accommodate TMI, the formation of binuclear sites is possible only with cations in the β-sites.^3^ It was previously shown that in the studied ferrierite matrix, the probability of the presence of two opposite β-rings containing Al pairs is above 95%.^5^ According to our best knowledge, besides the ferrierite employed, a suitable combination of structure and Al organization was reported only for mordenite with Si/Al 8.5.^9^ In other zeolite materials, the formation of a higher fraction of rings capable to accommodate binuclear sites and containing Al pairs was not determined. For example, in the case of the beta zeolite, a high fraction of opposite 6–MRs with Al pairs is observed only for samples with Si/Al 5 and ca. 55% of Al atoms in Al pairs in the β-sites.^4^ Thus, at least a qualitative analysis of the presence of four Al atoms capable to cooperate on the accommodation of binuclear sites formed by two cooperating divalent TMIs is of crucial importance for the development of systems for the activation of molecular oxygen for selective oxidation reactions.

According to our knowledge, there is no direct method for the analysis of the presence of two Al pairs in opposite zeolite rings or two cooperating divalent cations nor the spectroscopic difference between bare M(II) ions and two opposite M(II) ions in ca. 7 Å distance. This long distance, together with their rather low concentration and the complexity of the zeolite framework, makes binuclear TMI sites “invisible” for extended X-ray absorption fine structure or diffraction experiments. Up to now, a possible way to verify the presence of binuclear sites is performing the reaction test—splitting of molecular oxygen and subsequent reaction with methane.^4,5^ However, it is already reported that the luminescence of Cu(I) ions located in extra-framework cation sites of zeolite Y can be quenched by other TMI ions (Ni, Co, or Mn) when located close enough,^12^ which opens the possibility to develop a method for the analysis of the presence of two cations at a close distance. However, Cu(I) ions can be located in the vicinity of a single Al atom (not present in the Al-rich matrix of Y-zeolite); moreover, they can be mobile and thus do not represent a suitable probe for binuclear TMI sites active in methane oxidation.^13^ Our recent study showed that divalent Zn ions located in extra-framework positions of the ferrierite matrix (accommodated by Al pairs) exhibit similar luminescence properties as Cu(I) ions in zeolites and Zn(II) emission wavelength can be employed for the identification of Zn(II) siting.^14^ Therefore, the quenching of Zn(II) luminescence by other divalent cations of transition metal ions (M(II)) can be suggested as a possible way to prove the presence of two M(II) ions in close vicinity forming binuclear TMI sites. In this work, the introduction of Zn(II) (emitter) and Co(II) (quencher) to develop a method for studying the intrinsic property of the chosen FER matrix with Al distribution assuring the formation of binuclear catalytic centers with appropriate geometry,^5^ offering O_2_ splitting for selective oxidation reactions, is reported.

## Methods

In order to prepare the bimetallic samples, first, a commercially supplied ferrierite (FER) with Si/Al 8.5 (Tosoh Corporation, Japan), was exchanged with 1 M NH4NO_3_ (3 × 24 h at room temperature (RT)) and then exchanged again with 1 M of NaNO_3_ (3 × 24 h at RT). The obtained samples were washed with distilled water and dried in open air. Na-FER samples were further 3× exchanged using mixed solutions containing Zn (0.05 M zinc acetate) and Co (0.05 M cobalt nitrate) in various proportions (see Table 1). After washing slurries with distilled water, samples were dried in open air and calcined overnight in O_2_ flow at 450 °C.

**Table 1:** Parameters of Ionic Exchange and Chemical Composition of the ZnCo-FER Samples

	parameters of ionic exchange		chemical composition^a^					
sample	Zn (mL/g)	Co (mL/g)	Zn/Al	Co/Al	M/Al	Zn (mmol/g)	Co (mmol/g)	M (mmol/g)
ZnCo-FER-1	25	75	0.04	0.13	0.17	0.06	0.23	0.29
ZnCo-FER-2	50	50	0.07	0.12	0.19	0.13	0.21	0.34
ZnCo-FER-3	75	25	0.12	0.12	0.24	0.20	0.21	0.41

a Based on XRF results.

Chemical analysis of samples (see Table 1) was performed based on X-ray fluorescence spectra obtained on a BRUKER AXS S8 Tiger spectrometer. Measured data sets were evaluated using Spectra plus V3 software for the semiquantitative determination of elements from fluorine to uranium with a 5–10% error.

For analysis of the siting of bare Co(II) ions in extra-framework cationic sites of ferrierite, visible spectra of samples were collected using a PerkinElmer vis spectrometer Lambda 19 equipped with an integrating sphere for diffuse-reflectance measurements covered by Spectralon, which also served as a reference. Prior to the measurement granulated samples (0.3–0.6 mm fraction) were evacuated under dynamic vacuum (1 × 10^–3^ Pa) at 450 °C (4 °C/min heating ramp) for 3 h, transferred into an optical quartz cuvette, and sealed. The reflectance intensities were expressed using the Schuster–Kubelka–Munk eq 1:
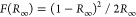
1where *R*∞ is the diffuse reflectance from a semi-infinite layer and *F*(*R*∞) is proportional to the absorption coefficient. Further spectra were simulated in Origin 8.1 software (OriginLab, Northampton, MA) and simulated using Gaussian bands assigned to the particular cationic position of cobalt in FER according to the method described elsewhere.^15,16^

For analysis of nature and siting of Zn ions, time-resolved luminescence spectra of dehydrated (3 h at 450 °C under dynamic vacuum) zeolites were recorded on an LKS 20 laser kinetic spectrometer (Applied Photophysics). The samples in a 5 mm thick silica cell were excited by a Lambda Physik COMPEX 102 excimer laser (wavelength, 308 nm; pulse width, 28 ns; pulse energy, ∼100 mJ). A long-pass filter (>330 nm) was situated between the cell and the monochromator. The emission signal at the selected wavelength was detected by the R928 photomultiplier, collected in a 600 MHz oscilloscope (Agilent Infiniium 54830B), and processed by a computer. The signal-to-noise ratio was improved by averaging 10–20 individual traces (depending on the emission intensity). All the measurements were carried out at room temperature. The emission spectra at selected times after excitation were constructed from the values of emission intensity at the individual wavelengths using Applied Photophysics Kinetic Spectrometer software. The lifetime of the corresponding emitting species is determined by the previous fitting of a double-exponential function
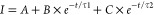
2to the experimental data of the luminescence decay of each species. I is the intensity of the luminescence signal, *t* is time (equal to 0 in the laser pulse maximum), and τ1 and τ2 are the excited-state lifetimes, whereas *A*, *B*, and *C* represent the fit parameters. The τ2 value was used to characterize the Zn(II) luminescence center. Qualitative and quantitative analysis of zinc in the studied samples was performed according to the studies presented elsewhere.^12^

As a complementary method for the confirmation of the introduction of divalent Co and Zn cations, FTIR spectroscopy was employed. Infrared spectra were recorded using an FTIR spectrometer (Nicolet 6700) equipped with a liquid-nitrogen-cooled MCTB detector. The quartz cell with KBr windows connected to a vacuum allowed the transport of the sample between the heating and measurement regimes. Thin, self-supporting pellets of a sample with an average density of 10 mg cm^–2^ were placed in the holder, and samples were evacuated before measurement under a dynamic vacuum (10^–3^ Pa) at 450 °C for 3 h. FTIR spectra were recorded at RT in the range between 4000 and 400 cm^–1^, with a single spectrum consisting of 128 scans at the resolution of 2 cm^–1^. Spectra were normalized on the density of the self-supporting pellets.

## Results and Discussion

The series of bimetallic ZnCo-FER samples were prepared (see the Methods section) and characterized in terms of their structural and spectroscopic properties. According to the data gathered in Table 1, the M/Al ratio (where M is a sum of Zn and Co) reaches ca. M(II)/Al 0.32.^9−11^ The total M/Al ratio in the range of 0.17–0.24 represents a good balance between the undesirable formation of other atomically dispersed bare M(II) metal ion species and reaching the alternating occupation of neighboring cationic sites (binuclear sites) with one Co(II) required for the study.^11,14,17^ The constant amount of Co mmol/g in studied samples (Table 1) suggests its higher tendency to be introduced to the zeolite during ion exchange in FER compared to Zn species.

**Figure 1:**
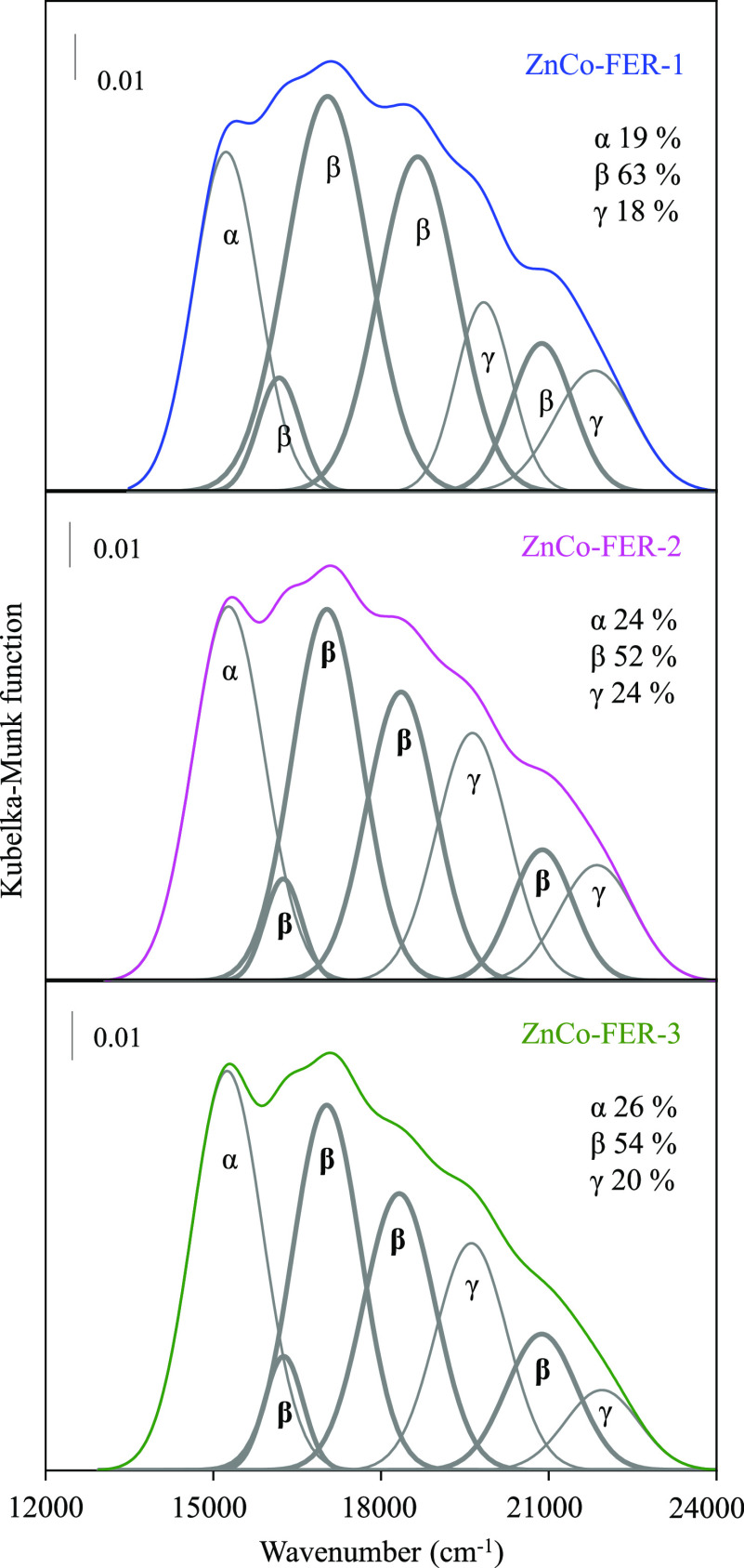
UV–vis spectra of the ZnCo-FER samples with the simulation of Co(II) in a particular cationic positions: α, β, and γ.

Visible spectra recorded for the ZnCo-FER samples were compared with Zn-FER to resolve the possible overlap of the bands from Zn(II) and Co(II). As no signals from Zn(II) are observable (see Supplementary Information, Figure S1) in this region (the Zn(II) cation exhibits a d^10^ electronic state; therefore, only d → s electron transitions are possible and their energy occurs at the UV region), visible bands were assigned to the Co(II) d–d transitions.^11^ Therefore, all three ZnCo-FER spectra were simulated into particular cationic positions of divalent Co and gathered in Figure 1. Qualitative and semi-quantitative analysis of those spectra based on the method established elsewhere^10,11,18,19^ (also see Methods) confirm that they represent features characteristic for Co-exchanged ferrierite with divalent metal species in the α, β, and γ positions.^11^ The charge-transfer band of the Co-oxo species was not observed, and thus, it was concluded that exclusively bare Co(II) species are present in the sample. The percentage value is the highest for Co(II) in the β site reaching 52–63%, which corresponds to 0.11–0.15 mmol total Co concentration (see Supplementary Information, Table S1). The α and γ cationic sites are much less populated and oscillating around 19–26 and 18–24%, respectively, corresponding to concentrations below 0.6 mmol.

For the analysis of the siting and distribution of divalent TMI in the extra-framework sites of zeolite, FTIR spectroscopy of antisymmetric skeletal T–O–T vibrations is often used.^14,17,20−22^ The accommodation of the cation results in the perturbation of the T–O–T vibrations and the appearance of the complex band at 1000–800 cm^–1^, which can be used for qualitative and quantitative analysis of the cations present in the extra-framework positions^21,22^ (such a band is not observed for the H- and Na-exchanged forms of zeolites).^17,23^ Positions of the particular bands of Co-FER and Zn-FER are published elsewhere.^14,17^ FTIR spectra records of the ZnCo-FER samples were compared with those of the Co-FER, Zn-FER and parent Na-FER samples and showed that due to the overlap in the frequency characteristic for Co and Zn^14,15,17^ (see Figure S2 in the Supporting Information), they cannot be distinguished and fitted into particular bands.

**Figure 2:**
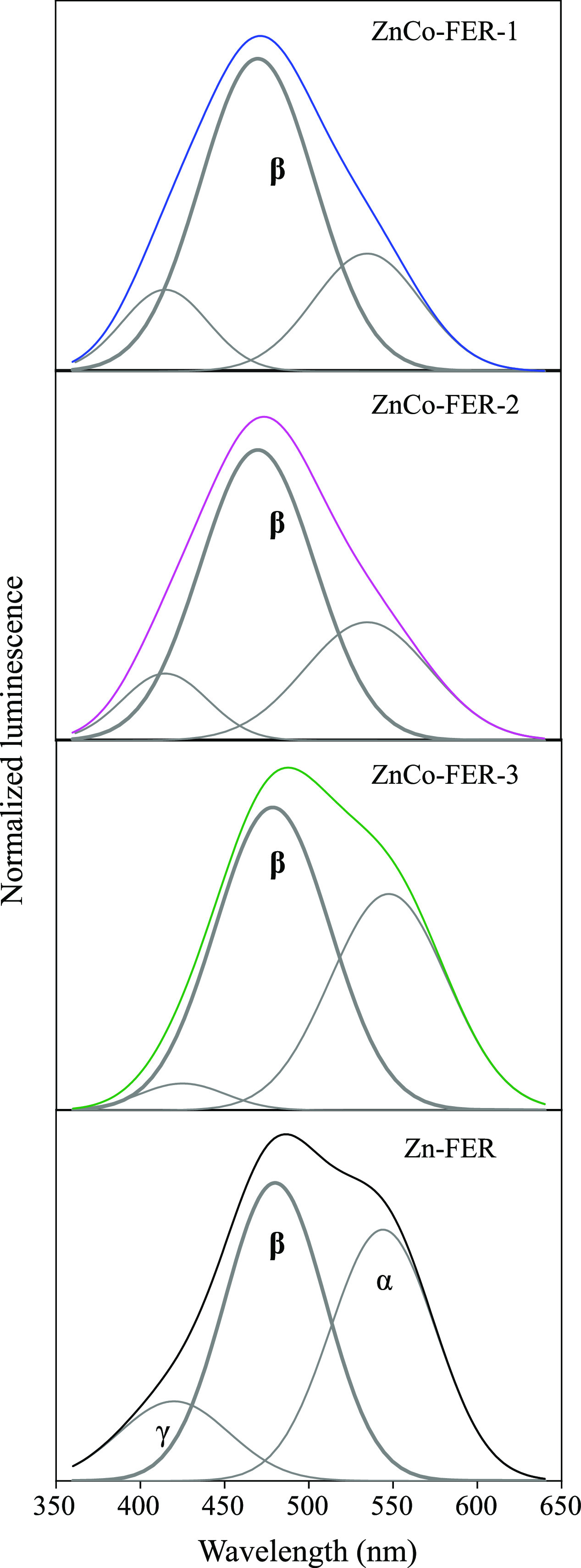
Normalized luminescence spectra of the ZnCo-FER samples and Zn-FER for comparison^14^ with the simulation of particular cationic positions: α, β, and γ.

To characterize the luminescence properties of the ZnCo-FER samples, emission spectra were acquired and gathered in Figure 2. They exhibit similar features to those already reported for Zn-exchanged ferrierite, with Zn(II) located in all cationic sites with maxima at 545, 480, and 425 nm for the α, β, and γ sites respectively, as reported in our previous work.^14^

**Figure 3:**
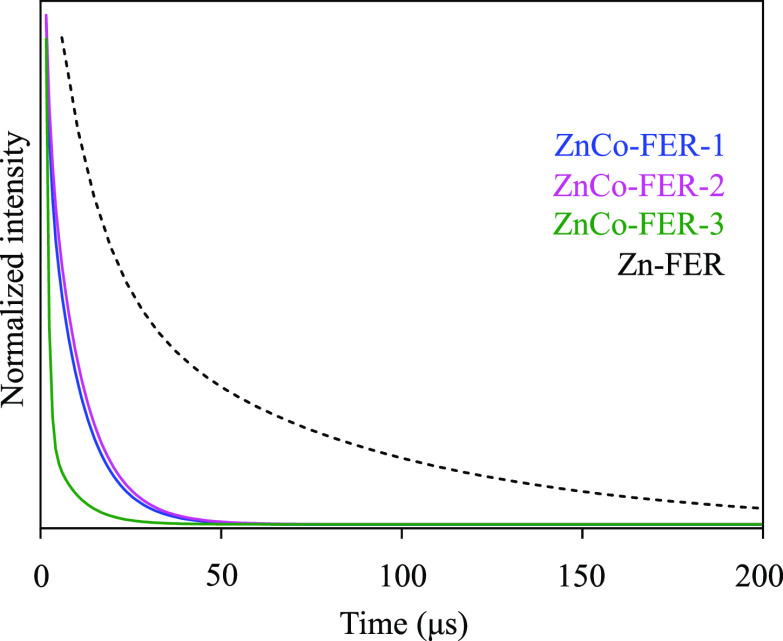
Decay of the Zn(II) emission in β-sites for the ZnCo-FER samples together with the decay of Zn-FER (dashed line) for comparison.

Although simulations of the particular cationic positions prove the presence of luminescent divalent zinc species, this method can be used in this particular case merely for qualitative analysis due to the presence of cobalt resulting in quenching. As shown in Figure 3, the introduction of Co(II) substantially decreases the emission lifetime in β-sites, and the effect is the most pronounced for the sample with the highest Zn/Co ratio—ZnCo-FER-3. The lifetime values obtained by the fitting of each sample (exponential decay), gathered in Table 2, confirm that it is also the case for all remaining cationic sites.

**Table 2:** Decay Times of Individual Luminescence Bands of the ZnCo-FER Samples and Zn-FER for Comparison and Critical Transfer Radius *R*_0_ Calculated According to Eq 4

sample	wavelength (nm)	lifetime τ_2_ (μs)	*R*_0_ (Å)
ZnCo-FER-1	545	11	14.5
	480	9	10.2
	425	8	12.9
ZnCo-FER-2	545	11	14.5
	480	10	10.0
	425	8	12.9
ZnCo-FER-3	545	10	14.8
	480	8	10.4
	425	6	13.6
Zn-FER	545	135	14.5
	480	65	10.2
	425	55	12.9

### Resonant Energy Transfer as a Probe of the Cation Vicinity

Several points concerning the mechanism type of the Zn(II) ion emission quenching in Zn-ferrierites need to be considered: (i) quenching by the collision mechanism, (ii) quenching via electron charge transfer to the quencher, and (iii) quenching by resonance energy transfer. The first one (point (i)) is well known for luminescence in solutions; however, in the dehydrated zeolites, atomically dispersed cations are stabilized in extra-framework cationic positions and their mobility is strictly limited. Thus, this type of luminescence quenching can be excluded as it is experimentally confirmed by the observed extremely long decay time of the Zn(II) emission of the bare cations.^14^ The luminescence quenching by charge transfer from the cation to the ligand (point (ii)) is well known for metallozeolites and was observed also for Zn(II) ions in the ferrierite.^12^ However, this mechanism of luminescence quenching can be excluded in the fully dehydrated Zn-zeolites and would be reflected in significantly more pronounced luminescence quenching (very short decay times of approximately 10 μs for Zn-ferrierite with guest molecules).^14^ Luminescence quenching of bare TMI in extra-framework cationic sites of the zeolite by resonance energy transfer (point (iii)) was investigated in the case of zeolite matrices for Cu(I) ions as emission centers and Co(II) and Ni(II), Co(II), and Mn(II) ions as quenchers located in the matrix of the Y-zeolite.^12^ As reported in works by Förster, this phenomenon can be explained by resonant energy transfer between the sensitizer (luminescence center; energy absorber) and activator (to which energy is transferred and which causes luminescence quenching), which fits well the experimental results.^12,24,25^ Due to the isoelectronic nature of the Cu(I) and Zn(II) ions, they exhibit quite similar luminescence properties, as was shown in luminescence/emission studies on Cu(I) and Zn(II) containing silicon-rich zeolites (emission bands at 540, 480, and 450 nm for Cu(I) and 540, 480, and 425 nm for Zn(II) and luminescence decays ranging from 120–35 and 135–55 for Cu(I) and Zn(II), respectively).^14,26^ Therefore, conclusions for Cu(I) can be transferable also to Zn(II) luminescence/quenching in the presence of Co(II) (detailed discussion of the application of Förster–Dexter theory to the quenching of metal ion luminescence in zeolites, see ref (12)).

A non-radiative resonant energy transfer causing luminescence quenching of the bare Zn(II) ions in extra-framework cationic sites of the zeolite by other divalent transition metal ions requires fulfilling two conditions: (i) close vicinity of emitting and quenching cations shorter than the so-called critical transfer radius and (ii) close energy of the quenched triplet state of the Zn(II) ion and the singlet state of the quenching cation.

**Scheme 1:**
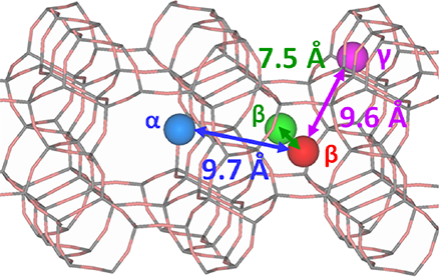
Schematic Representation of M(II) Cationic Sites in Ferrierite Together with the Shortest Possible Distances between Co(II) in the β-Sites (Red) and Zn(II) in the α- (Blue), β- (Green), and γ-Sites (Violet)

For point (i), in the case of zeolite of ferrierite topology, three extra-framework cationic sites, denoted as α-, β-, and γ-sites. are known to accommodate bare divalent metal ions. The local arrangement of cationic sites, the positions of cations in these sites, and the distances between cations in the individual sites are gathered in Scheme 1.^9,11,27,28^ Distances between M(II) ions in all three cationic sites are significantly smaller than the critical transfer radius observed earlier (i.e., limiting distance at which quenching is no longer observed; 13–25 Å), which suggests that Zn(II) ions in these sites can be quenched by the neighboring M(II) ion. For investigated ferrierite, the β-sites predominate, while the α- and γ-sites are barely populated (based on literature reports of 1, 0.2, and 0.06 M(II) in the β-, α-, and γ-sites per unit cell, respectively, calculated based on references).^11^ Thus, a significant presence of the quenching M(II) ion in the β-sites is required for an observable effect on the Zn(II) luminescence (note that the M(II) ion in the β-sites can, due to its high concentration, also quench Zn(II) ions in the α- and γ-sites, while the opposite situation—quenching of Zn(II) ions by the M(II) ion in the α- and γ-sites—is of low probability).

For point (ii), the close energy of luminescence and absorption energy levels of the cations is reflected in the overlap of the luminescence spectrum of Zn(II) ion (s^1^d^9^ → d^10^ transition) and absorption of the M(II) transition metal ion quencher (d–d transition).

To allow observable quenching of the Zn(II) luminescence, a high population of quenching Co(II) ions is required. As follows from the analysis of the Co(II) siting in all investigated ZnCo-ferrierite samples (Table 1), Co(II) ions are predominantly present in the β-sites, and all conditions required for the non-radiative resonance quenching of the bare Zn(II) ion located in the extra-framework cationic site of ferrierite is fulfilled.

**Figure 4:**
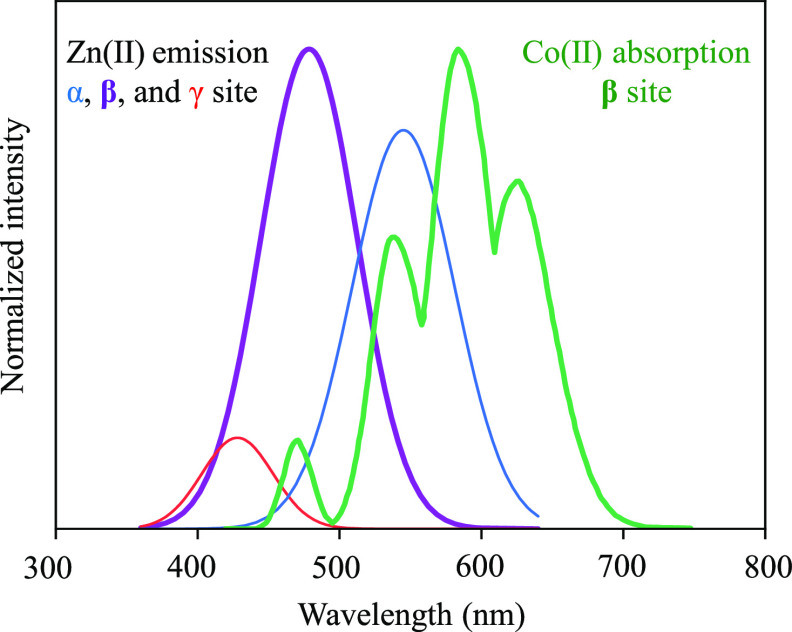
Overlap of the Zn(II) emission bands in the α-, β-, and γ-sites and Co(II) absorption in the β-site.

Emission spectra of all ZnCo-ferrierites exhibit three luminescence bands at 425, 480, and 545 nm.^14^ On the contrary, Zn(II) emission lifetimes are significantly shorter in ZnCo samples compared to the Zn(II) ones. This indicates that all three cationic sites of the ferrierite matrix are occupied by Zn(II) ions and their emission is quenched. Note that quenching of Zn(II) luminescence by other Zn(II) ions was not observed (and is in discrepancy with the Förster–Dexter theory) even at the highest Zn loadings significantly exceeding the loading in ZnCo samples,^14^ excluding the quenching of Zn(II) luminescence by resonance transfer to other Zn(II) ions. Also, non-radiative resonance energy transfer to other M(II) ions in the zeolite caused by the adsorption of guest molecules (O_2_ or H_2_O)^14^ can be ruled out as it would be connected with a more pronounced decrease in the luminescence lifetime than that observed for the dehydrated ZnCo-ferrierite samples as mentioned above. To confirm the resonant energy transfer nature of the quenching between Zn(II) and Co(II), the overlap of emission and absorption bands was experimentally investigated and shown in Figure 4 for sample ZnCo-FER-3. The Co(II) absorption band in the β-site is broad enough to ensure the possibility of energy transfer between the α-, β-, and γ-sites of the emitter and the β-site of the quencher (α–α, α–γ, and γ–γ interactions were neglected due to the significantly longer distances between them, lowering the possibility of the transfer). Therefore, as the energy resonance transfer corresponds to the observed quenching of Zn(II) luminescence, its quenching stands as evidence of the vicinity of two bare M(II) cations, i.e., the formation of distant binuclear sites.

Estimation of the Zn(II)–Co(II) critical transfer radius can be suggested as final confirmation of the Zn(II) luminescence quenching via energy resonance transfer and, thus, the formation of binuclear sites. According to the Förster–Dexter theory,^12,29^ the rate of energy transfer *k*_T_ is given by eq 3:
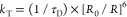
3where *R*_0_ is the critical transfer radius, *τ*_D_ is the donor (Zn(II)) lifetime in the absence of the acceptor (Co(II)), and *R* is the distance separating the Zn(II) and Co(II). The parameter *R*_0_ is defined as the acceptor–donor separation radius for which the transfer rate equals the rate of donor decay (de-excitation) in the absence of the acceptor. The efficiency of energy transfer, ET (the fraction of photons absorbed by the donor that are transferred to the acceptor), is related to *R* by eq 4:
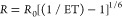
4

ET can be evaluated as (eq 5):
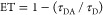
5where τ_DA_ is the donor’s lifetime in the presence of the acceptor. Therefore, by measuring the donor luminescence lifetime in the presence and absence of an acceptor and the known distance separating the donor and acceptor molecules (Scheme 1), it is possible to calculate the value of *R*_0_ according to eq 6:
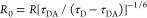
6where τ_DA_ is the lifetime τ_2_ of ZnCo-FER in Table 2, τ_D_ is the lifetime τ_2_ of Zn-FER in Table 2, and *R* is the distance between the cation in the β-site and the neighboring α-, β-, and γ-sites given in Scheme 1.

However, the available experimental data can be employed only for a rough estimation of the critical energy transfer radius between Zn(II) and Co(II) ions using eq 4 due to several reasons: (i) The real mutual arrangement of Zn(II) and Co(II) ions in the α-, β-, and γ-sites can overlap due to the low-framework Al content having several arrangements with different inter-cationic distances as reflected in the two-exponential dependence of the luminescence decay, indicating more complex situation than that corresponding to the Förster–Dexter theory (giving single-exponential luminescence decay). (ii) Data cover only intercationic distances between 7.5 and 9.7 Å. The value of critical transfer radius *R*_0_ = 13 ± 2 Å calculated from data in Table 2 and Scheme 1 fits well with the values reported earlier for resonance energy transfer from Cu(I) cations to divalent transition metal ions (15 Å for Ni(II), 22 Å for Co(II), and 13 Å for Mn(II) at higher M(II) loadings).^12^ This sound agreement between values observed for the Zn(II) ion and isoelectronic Cu(I) further confirms that quenching of the Zn(II) ions luminescence in dehydrated ZnCo-ferrierites occurs via an energy-transfer mechanism. The resonance energy transfer from the Zn(II) ion in the extra-framework cationic position to the Co(II) one suggests that there is a short distance between the Zn(II) and Co(II) ions in the zeolite. Such nonradiative energy transfer confirms the presence of the Zn(II)–Co(II) binuclear TMI sites in the investigated samples with a given ferrierite matrix.

The available methodology based on the application of divalent cations allowed us to only analyze the siting and concentration of single Al atoms or Al pairs using M(II) ions as probes monitored by FTIR of the UV–vis spectra.^9,10^ The only reasonable conclusions about the presence of four cooperating Al atoms in the zeolite framework can thus be made for zeolites in which high occupation of cationic sites by divalent cations cannot be reached without the close distance between occupied cationic sites, as was already shown for ferrierite zeolite with Si/Al 8.5 and 60% of Al in Al pairs.^5^

On the other hand, the methodology of analysis of Al organization in the framework based on Zn(II) emission quenching can be extended to other materials with a framework (or network) bearing negative charges balanced by extra-framework cationic species such as zeolite analogues with other isomorphously substituted Al atoms, e.g., boralites or gallium zeolites,^30,31^ zeotypes (e.g. metalloaluminophosphates),^32^ aluminosilicate mesoporous molecular sieves,^33^ or amorphous analogues of zeolites such as geopolymers/alkali-activated ceramics.^34−36^ All these materials are applied or represent promising materials in the field of catalysis.^34−36^ and studies on the organization of frameworks that control the siting and distribution of active sites are issues of significant importance.

The presence of four framework Al atoms arranged as two Al pairs (Al–O–(Si–O)_2_–Al sequences) in two different close zeolite rings was for the first time directly confirmed by a newly developed method, enabling us to evaluate the possibility of the distant binuclear transition metal ion sites formation in the zeolite matrix. This method is based on the luminescence quenching of the bare Zn(II) in the extra-framework cationic sites by the presence of Co(II) co-cations in the dehydrated CoZn-zeolite matrix. For CoZn-ferrierite with the known capability to accommodate distant binuclear TMI sites, it was demonstrated that the luminescence of Zn(II) cations located in all three extra-framework cationic sites for divalent cations is quenched when the Co(II) ions are predominantly located in the close-distance β-site. The mechanism of Zn(II) emission quenching by energy resonance transfer is independent of the topology of the zeolite framework and controlled only by the cation distance in the zeolite. Thus, it can be employed to investigate the distances of the cationic sites of divalent cations (zeolite 6- or 8-MR rings with Al pairs) in zeolites and related materials. As mentioned in the Introduction, activation of molecular oxygen by splitting can be regarded as a general property of two divalent transition metal ions in a proper arrangement and is a base for the development of highly active and selective catalysts.

## Conclusions

The presence of four framework Al atoms arranged as two Al pairs (Al–O–(Si–O)_2_–Al sequences) in two different close zeolite rings was for the first time directly confirmed by a newly developed method, enabling us to evaluate the possibility of distant binuclear transition metal ion site formation in the zeolite matrix. This method is based on the luminescence quenching of the bare Zn(II) in extra-framework cationic sites by the presence of Co(II) co-cations in the dehydrated CoZn-zeolite matrix. For CoZn-ferrierite with a known capability to accommodate distant binuclear TMI sites, it was demonstrated that the luminescence of Zn(II) cations located in all three extra-framework cationic sites for divalent cations is quenched when Co(II) ions are predominantly located in the close-distance β-site. The mechanism of Zn(II) emission quenching by energy resonance transfer is independent of the topology of the zeolite framework and controlled only by the cation distance in the zeolite. Thus, it can be employed to investigate the distances of the cationic sites of divalent cations (zeolite 6- or 8-MR rings with Al pairs) in zeolites and related materials. As mentioned in the Introduction, activation of molecular oxygen by splitting can be regarded as a general property of two divalent transition metal ions in a proper arrangement and is a base for the development of highly active and selective catalysts. Unquestionable confirmation or exclusion of the presence of two Al pairs in opposite rings in the zeolite is complex, difficult, and time-demanding. Compared with the reaction tests, analysis of Al organization in the framework through quenching of Zn(II) emission is universal, simple, and time-saving and can significantly contribute to the screening process of searching for a suitable catalyst for methane oxidation and other important oxidation reactions.

## Supporting Information Available

(Figure S1) UV–vis spectra of ZnCo-FER samples together with the spectrum of Zn-FER (dashed line) shown for comparison; (Figure S2) FTIR spectra of the ZnCo-FER samples in the range of T–O–T vibrations together with Na-FER (dashed line) for comparison; (Table S1) Co(II) distribution in the α, β, and γ cationic sites (PDF)
