# Supplementary material for: Organization of Cooperating Aluminum Pairs in Ferrierite Evidenced by Luminescence Quenching
Source: J Phys Chem C Nanomater Interfaces. 2023 Apr 7;127(15):7344–51. doi: 10.1021/acs.jpcc.3c00585 (PMC10123814; doi:10.1021/acs.jpcc.3c00585)
Supplement: Supplementary file 1 — jp3c00585_si_001.pdf [file jp3c00585_si_001.pdf]

Supporting Information for

Organization of Cooperating Aluminum Pairs in Ferrierite Evidenced by Luminescence Quenching

Joanna E. Olszowka, Pavel Kubat, Jiri Dedecek, Edyta Tabor*

J. Heyrovský Institute of Physical Chemistry of the Czech Academy of Sciences, Dolejškova 2155/3, 18200, Prague, Czech Republic

* corresponding author; e-mail: edyta.tabor@jh-inst.cas.cz

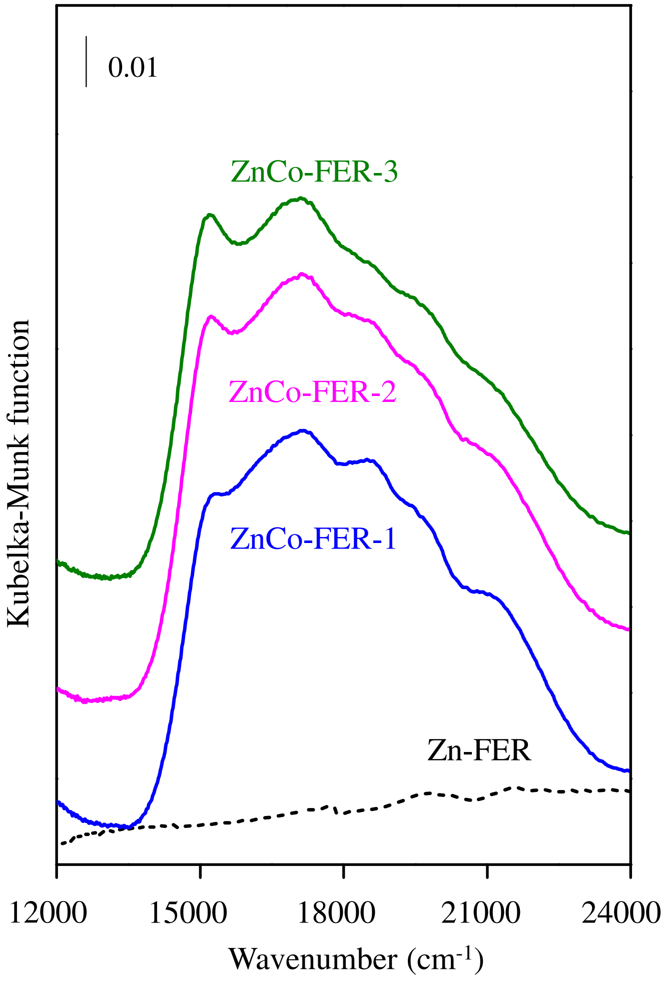

Figure S1. UV-Vis spectra of ZnCo-FER samples together with the spectrum of Zn-FER (dashed line) shown for comparison.

Table S1. Co(II) distribution in α, β, and γ cationic sites.

Sample	Cationic sites					
	α	β	γ	α	β	γ

	%*			mmol#		
ZnCo-FER-1	19	63	18	0.04	0.15	0.04
ZnCo-FER-2	24	52	24	0.05	0.11	0.05
ZnCo-FER-3	26	54	20	0.06	0.11	0.04

*based on Vis spectra #calculated from % and chemical analysis

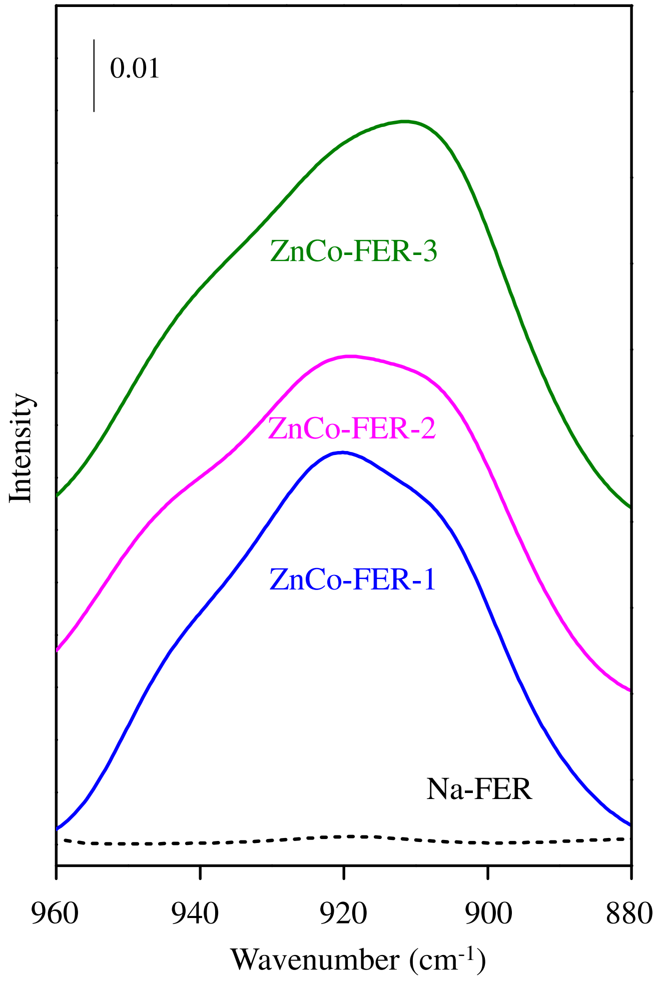

Figure S2. FTIR spectra of the ZnCo-FER samples in the range of T-O-T vibrations together with Na-FER (dashed line) for comparison.
